# A new species of *Pista* Malmgren, 1866 (Polychaeta, Terebellidae) from the north-western Mediterranean Sea

**DOI:** 10.3897/zookeys.838.28634

**Published:** 2019-04-11

**Authors:** Céline Labrune, Nicolas Lavesque, Paulo Bonifácio, Pat Hutchings

**Affiliations:** 1 Sorbonne Universités, CNRS, Laboratoire d’Ecogéochimie des Environnements Benthiques, LECOB UMR 8222, F-66650 Banyuls-sur-Mer, France Sorbonne Universités Banyuls-sur-Mer France; 2 University of Bordeaux, EPOC, UMR 5805, Station Marine d’Arcachon, 2 Rue du Professeur Jolyet, 33120 Arcachon, France University of Bordeaux Arcachon France; 3 CNRS, EPOC, UMR 5805, Station Marine d’Arcachon, 2 Rue du Professeur Jolyet, 33120 Arcachon, France CNRS, EPOC Arcachon France; 4 Ifremer, Centre Bretagne, REM EEP, Laboratoire Environnement Profond, ZI de la Pointe du Diable, CS 10070, F-29280 Plouzané, France Ifremer, Centre Bretagne, REM EEP, Laboratoire Environnement Profond Plouzané France; 5 Australian Museum Research Institute, Australian Museum, 1, William Street, Sydney, NSW 2010, Australia Australian Museum Research Institute, Australian Museum Sydney Australia; 6 Department of Biological Sciences, Macquarie University, North Ryde 2109, Australia Macquarie University North Ryde Australia

**Keywords:** Annelida, gravel deposits, harbour, *Pistacolini* sp. n., taxonomy, Terebellida

## Abstract

A new species of Terebellidae, *Pistacolini***sp. n.**, has been identified from the harbour of Banyuls-sur-Mer, north-western Mediterranean Sea. This new species was found in very high densities, exclusively in gravelly sand deposited manually, and was not found in the original source habitat of the gravel. This species is characterized by the colour of the ventral shields with pinkish anterior part and a blood red posterior part in live specimens, a pair of unequal-sized plumose branchiae inserted on segment II and anterior thoracic neuropodia with long-handled uncini. The presence of long-handled uncini even in the smallest specimens constitutes the major difference between *Pistacolini***sp. n.** and other *Pista* species with a single pair of branchiae such as *P.lornensis* and *P.bansei*.

## Introduction

Terebellids belong to a very species-rich group of sedentary polychaetes, widely distributed in most marine benthic substrates, from shallow waters to deep-sea environments (Hutchings 2000, Rouse and Pleijel 2001). A recent review of Terebellidae Johnston, 1846 has been undertaken by Hutchings et al. (2017). The genus *Pista* Malmgren, 1866 currently includes 74 valid species (Hutchings et al. 2017). Difficulties in observing morphological characters and lack of geographically relevant literature have led to misidentifications of specimens belonging to this group. For example, *Pistacristata* (Müller, 1776) has been considered as a cosmopolitan species, but represents a complex of species (Gil 2011, Hutchings and Kupriyanova 2018). Recently, many changes have occurred in the *Pistasensu lato* group (Hutchings et al. 2017, Jirkov and Leontovich 2017) which currently includes seven genera: *Axionice* Malmgren, 1866, *Eupistella* Chamberlin, 1919, *Lanicides* Hessle, 1917, *Paraxionice*, Fauchald, 1972, *Pista* Malmgren, 1866, *Pistella* Hartmann-Schröder, 1996, and *Scionella*Moore 1903. Among these genera, only four have some species with a single pair of branchiae: *Pista*, *Lanicides*, *Pistella*, and *Scionella*. *Pista* and *Lanicides* can be differentiated from each other by the shape of avicular uncini and particularly by the presence of long-handled uncini (Hutchings et al. 2017). Nogueira et al. (2010, 2015) highlighted the presence of distally serrated notochaetae in *Lanicides*, which are absent in *Pista*. Furthermore, according to Nogueira et al. (2010), species of *Scionella* have a single pair of branchiae on segment IV while those of *Pistella* have a single pair of branchiae on segment II. Mikac and Hutchings (2017) provide a generic diagnosis of *Pistella* versus *Pista.* According to them, the main difference between these two genera is that *Pistella*’s neurochaetae are all short-handled avicular uncini while *Pista*’s neurochaetae are long-handled avicular uncini, at least on some anterior neuropodia.

Currently, morphological-based studies on *Pista*-like genera (Saphronova and Jirkov 2001; Gil 2011) consider the number of pairs of branchiae and the presence of long-handled anterior thoracic uncini as size-related characters, and therefore synonymized several genera and suggested some species have very wide distributions. However, it is clear that a detailed revision of all these genera is required using both morphological and molecular techniques. Ontogenetic studies could also clarify if the development of the long-handled uncini present in anterior thoracic neuropodia is a size-related character or is fixed for a species within a genus. Hutchings et al. (2017) and the present study accept them as stable generic characters and therefore reject these synonymies of Saphronova and Jirkov (2001). Currently, only seven species in the genus *Pista* are characterised by possessing a single pair of branchiae: *P.dibranchis* Gibbs, 1971 known from the Solomon Islands, *P.godfroyi* (Gravier, 1911) and *P.spinifera* (Ehlers, 1908) from Antarctica; *P.mirabilis* McIntosh, 1885 from deep water off Argentina; *P.bansei* Saphronova, 1988 described from Northern Pacific Ocean (although no specific type locality is given in the original description, but recently confirmed by Hutchings unpublished data.); *P.lornensis* (Pearson, 1969) from a Scottish loch, and finally *P.adriatica* Mikac & Hutchings, 2017 recently described from the Adriatic Sea.

The present study provides the description of a new species of *Pista* from the north-western Mediterranean Sea, based on morphological characters. Molecular data (COI gene) are provided for further investigations.

## Materials and methods

### Sampling and morphological analyses

**Figure 1.**
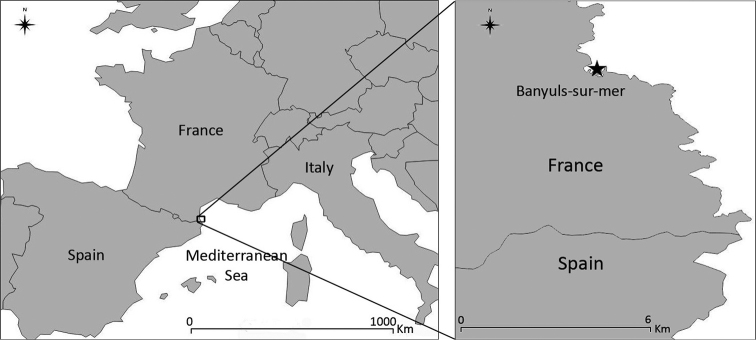
Location of Banyuls-sur-Mer harbour, France, where *P.colini* sp. n. was collected.

The first specimens of the new *Pista* species were sampled in 2012 in the harbour of Banyuls-sur-Mer (French Mediterranean Sea; WGS84: 42°28.87'N, 3°08.15'E; 3 m depth; Fig. 1). Specimens examined in this study were collected in 2012 and 2017 using a van Veen grab. Live specimens (anaesthetised with menthol) were examined under a Zeiss stereomicroscope (V20 discovery-Plan S objective 1.0×) equipped with a camera (Axiocam 105) and preserved specimens with a Nikon SMZ25 stereomicroscope (Nikon DS-Ri 2) camera, a Nikon Eclipse E400 microscope, and a Zeiss Axio Lab.A1 microscope. Slides for uncini were prepared with lactic acid and observed under 100× oil immersion lens. A posterior parapodium of paratype MNHN-IA-TYPE 1853 was removed and fixed in 100% ethanol for molecular studies. All other material was fixed in 4% formaldehyde seawater solution, then transferred to 70% ethanol for morphological analyses. Several specimens were dehydrated in ethanol, critical point dried and covered with gold, and examined under a scanning electron microscope (SEM) at Macquarie University (JEOL JSM 6480LA) and at Arcachon Marine Station (Hitachi TM3030).

Holotype and most paratypes were deposited at the Museum national d’Histoire naturelle, Paris (**MNHN**), other paratypes were deposited in the Australian Museum, Sydney (**AM**). Non-type additional material was lodged in collections of Banyuls-sur-Mer and Arcachon Marine Stations in France.

### DNA isolation, amplification, and sequencing

Samples for DNA analysis were removed from a live specimen (paratype MNHN-IA-TYPE 1853) placed in ethanol 96% and frozen at -20 °C. Extraction of DNA was done with QIAamp DNA Micro Kit (QIAGEN) following protocol supplied by the manufacturers. Approximately 650 bp of COI (cytochrome c oxidase subunit I) genes were amplified using primers polyLCO and polyHCO (Carr et al. 2011). The PCR (Polymerase Chain Reaction) was carried out with Gotaq G2 Flexi DNA Polymerase (PROMEGA), with 50 µL mixtures contained: 10µL of 5X Colorless GoTaq Reaction Buffer (final concentration of 1X), 1.5 µL of MgCl_2_ solution (final concentration of 1.5mM), 1 µL of PCR nucleotide mix (final concentration of 0.2 mM each dNTP), 0.5 μl of each primer (final concentration of 1µM), 0.2 µl of GoTaq G2 Flexi DNA Polymerase (5U/µl), 1 μl template DNA and 33.8 µL of nuclease-free water. The temperature profile was as follows: 94 °C/600s – (94 °C/40s-44 °C/40s-72 °C/60s) *5 cycles -(94 °C/40s-51 °C/40s-72 °C/60s) *35 cycles -72 °C/300s -4 °C. Amplified PCR products were analysed by electrophoresis in a 1 % p/v agarose gel stained with ethidium bromide and were sent to GATC Biotech Company to complete double strain sequencing, using same set of primers as used for PCR. Overlapping sequence (forward and reverse) fragments were merged into consensus sequences and aligned using Clustal Omega. Sequences were translated into amino acid alignment and checked for stop codons to avoid pseudogenes. The minimum length coverage was around 660 bp. Sequence obtained in this study has been deposited in GenBank (http://www.ncbi.nlm.nih.gov/genbank/). The accession number is given in the section Genetic data.

## Taxonomic account

### Family Terebellidae Johnston, 1846

#### Genus Pista

Taxon classificationAnimaliaTerebellidaTerebellidae

Malmgren, 1866

##### Type species.

*Amphitritecristata* Müller, 1776, by original designation.

##### Diagnosis.

Transverse prostomium attached to dorsal surface of upper lip; basal part as thick crest, eye spots sometimes present; distal part shelf-like. Buccal tentacles all uniformly cylindrical. Peristomium restricted to lips; relatively short upper lip, hood-like; swollen, cushion-like and mid-ventral lower lip. Segment I reduced dorsally, with pair of lobes of variable size and position; segments II–IV also with pairs of lobes of variable size and position, sometimes extending for a few more segments. Anterior segments highly glandular ventrally, with discrete, smooth to slightly corrugated, rectangular to trapezoidal mid-ventral shields. Paired arborescent, pectinate or plumose branchiae present from segment II, typically two pairs, on segments II and III, rarely a single pair or three pairs. Conical to rectangular notopodia beginning on segment IV, all aligned, typically extending for 17 segments, until segment XX; notochaetae all distally winged, frequently broadly winged. Neuropodia beginning on segment V, as low ridges in conjunction with notopodia and short pinnules posteriorly; neurochaetae as long-handled avicular uncini, at least on anterior neuropodia, frequently until segment X or termination of notopodia, then short-handled; uncini in partial to completely intercalated double rows on segments XI–XX. Nephridial papillae present on segment III, genital papillae on variable number of segments, usually on segments VI–VII, posterior and dorsal to notopodia. Pygidium smooth to slightly crenulated (after Hutchings et al. 2017).

#### Pista colini sp. n.

Taxon classificationAnimaliaTerebellidaTerebellidae

http://zoobank.org/0532761D-4534-4C83-8D56-D7683468160B

Figs 2 , 3 , 4

##### Material examined.

***Type material.*** Banyuls-sur-Mer harbour, Gulf of Lion, Mediterranean Sea, France (42°28.867'N, 3°08.154'E, 3 m depth), subtidal in gravely sands, all collected 16 July 2012 except MNHN-IA-TYPE 1853 collected 12 July 2017. Holotype: MNHN-IA-TYPE 1850, complete, 70 segments, total length 17.6 mm, thoracic length 4.8 mm, anterior width 0.6 mm, Paratypes: AM W.50625, 1 specimen, posteriorly incomplete, total length 11 mm, thoracic length 7 mm, anterior width 1.0 mm; AM W.50626, 3 specimens plus 1 posterior fragment 5 mm with pygidium, 1 complete, total length 11 mm, thoracic length 5 mm, anterior width 0.5 mm, 1 complete, total length 12 mm, thoracic length 5 mm, anterior width 0.5 mm, 1 posteriorly incomplete, length 16 mm, thoracic length 8 mm, anterior width 0.8 mm, 2 specimens mounted for SEM. MNHN-IA-TYPE 1851, 1 specimen, posteriorly incomplete, total length 14.3 mm, thoracic length 3.7 mm, anterior width 0.7 mm; MNHN-IA-TYPE 1852, complete specimen, total length 9.70 mm, thoracic length 4.6 mm, anterior width 0.9 mm; MNHN-IA-TYPE 1853, complete collected 12 July 2017, thoracic length 4.4 mm, anterior width 1.1 mm, posterior part cut for molecular analysis; MNHN-IA-TYPE 1854, complete length 18.2 mm, anterior width 0.7 mm, mounted for SEM; MNHN-IA-TYPE 1855, complete, 1 specimen, total length 9.0 mm, thoracic length 3.1 mm, anterior width 0.7 mm.

***Additional material.*** Banyuls-sur-Mer harbour, Gulf of Lion, Mediterranean Sea, France (42°28.867'N, 3°08.154'E, 3 m depth), subtidal in gravely sands, all collected 16 July 2012. BAN.Pista.08, 1 specimen, complete, total length 10.0 mm, thoracic length 3.7 mm, anterior width 0.8 mm; BAN.Pista.09, 1 specimen gravid, posteriorly incomplete, thoracic length 5.1 mm, anterior width 0.7 mm; BAN.Pista.10, complete, 1 specimen, total length 22.7 mm, thoracic length 7.8 mm, anterior width 0.9 mm; BAN.Pista.12, complete, 1 specimen, total length 12.4 mm, thoracic length 4.6 mm, anterior width 0.6 mm.

***Comparative material.****Pistabansei* Saphronova, 1988 Holotype reg. # 47667, 47°41'N, 139°34.1'E, Sea of Japan, Tartary Strait, off Nelma, 105 m; 4 paratypes reg. # 47668 according to Saphronova 1988 (but reg. # 32423 according to label in the museum vial) from same station, deposited in Zoological Museum of Russian Academy of Sciences in St Petersburg.

Additional material from R/V “Vityaz” stations 59, 119, 1587a, 3350, 3569, 1086. (For locality details see Saphronova (1988: table 1, no museum registration numbers allocated) deposited in the Zoological Museum of Moscow State University.

##### Description

**Figure 2.**
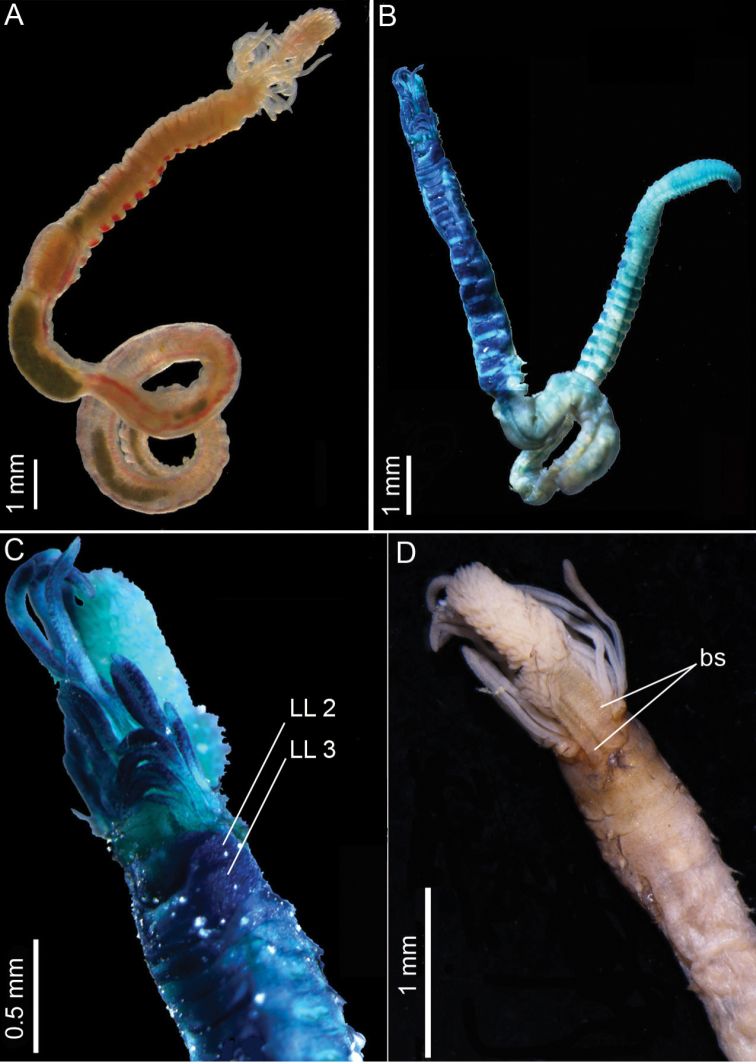
*Pistacolini* sp. n.: **A** Live specimen, dorsal view **B** Entire specimen, ventral view, methyl green staining **C** Anterior part, ventral view **D** Anterior part, dorsal view. **B–D** from holotype MNHN-IA-TYPE 1850. Key: LL: lateral lobes, bs: branchial stalks.

(based on holotype). Holotype is a complete specimen, 17.5 mm in length, 0.6 mm in width at segment X and with 70 segments (Fig. 2A, B).

**Figure 3.**
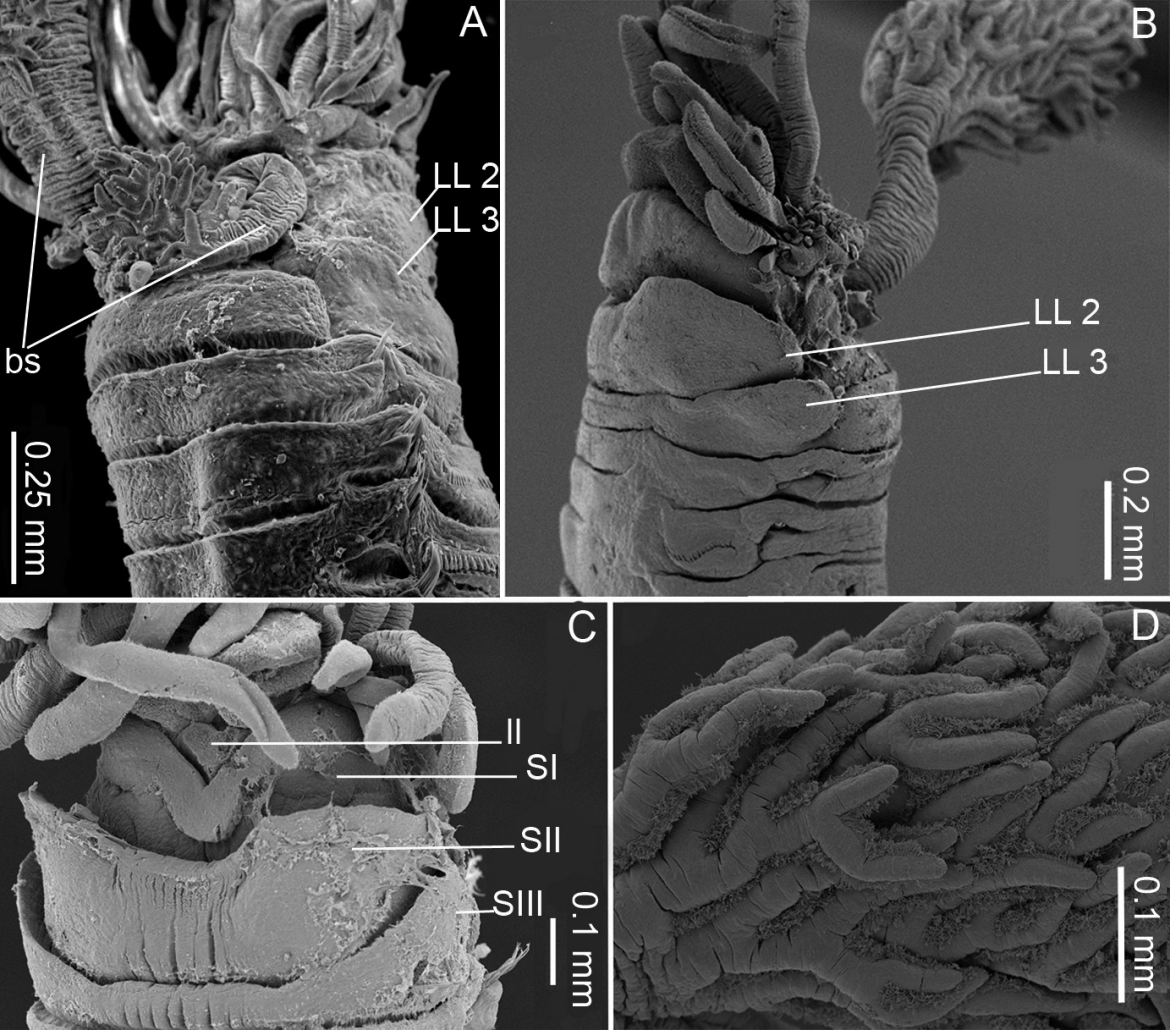
*Pistacolini* sp. n., SEM images: **A** Anterior part, dorso-lateral view **B** Anterior part, lateral view **C** Anterior part, ventral view **D** Branchial filaments **A** from paratype MNHN-IA-TYPE 1854 **B–D** from paratype AM W.50626. Key: LL: lateral lobes, bs: branchial stalks, ll: lower lip, SI, SII, and SIII: Segments I, II, and III.

Transverse prostomium attached to dorsal surface of upper lip. Buccal tentacles all of similar width inserted ventrally on prostomium, shorter than smallest branchia; long tentacles situated centrally in dorsal region, longer than largest branchia (Fig. 2C). Peristomium consisting of large rounded upper lip, forming a swollen cushion with one small fold on each side. Lower lip short, irregularly swollen (Fig. 3C). Segment I reduced, V-shaped, situated medio-ventrally (Fig. 3C), without lateral lobes. Segment II with well-developed lateral lobes, with anterior margins rounded merging with ventral pad to form a continuous minutely crenulated ventral collar. One pair of unequal-sized plumose branchiae inserted one just next to the other on segment II; all filaments strongly ciliated (Figs 2D, 3D), arranged in spiral around central axis with dichotomous filaments. Both stalks markedly wrinkled (Fig. 3A, B). Segment III with lateral lobes half width of segment, asymmetrical and slightly displaced dorsally, connected across ventrum (Fig. 3A–C). Segment IV lacking lateral lobes (Fig. 3A, B).

**Figure 4.**
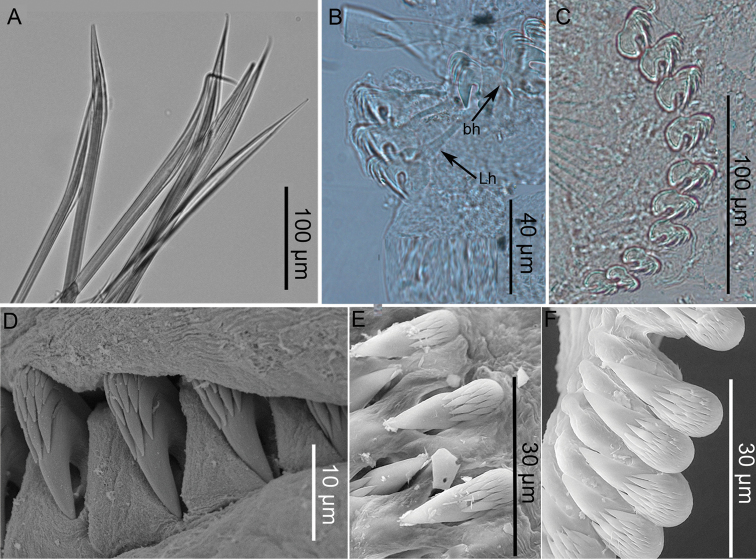
*Pistacolini* sp. n.: **A** Thoracic notochaeta of segment VI **B** Thoracic uncini of segment V **C** Abdominal uncini **D** Thoracic uncini in single row **E** Thoracic uncini in double row **F** Abdominal uncini **A** from paratype MNHN-IA-TYPE 1853 **B, C** from additional material BAN.Pista.12 **D** from paratype AM W.50626 (SEM image) **E, F** from paratype MNHN-IA-TYPE 1854 (SEM images). Key: Lh: long-handled uncinus, bh: broken-handled uncinus.

Notochaetae, broad-winged capillaries, with fine tips (Fig. 4A). Neuropodia from segment V (chaetiger 2), initially arranged in single rows, from segments XI to XX arranged in completely intercalated double rows face-to-face and then reverting to single rows on abdomen. Neurochaetae as long-handled avicular uncini on segments V and VI (Fig. 4B) then short-handled. Neuropodia with ca. 14 uncini (arranged in single row), thoracic uncini with dental formula MF: 3–4:5–6:α (Fig. 4D–E). Abdominal neuropodia becoming more erect posteriorly with ca. 12 uncini each, elongate extending from torus, dental formula MF: 6–7:6–7: α: α (Fig. 4C, F). Nephridial papillae on segments VI and VII (chaetigers 3 and 4), inserted posteriorly/laterally to notopodia, small spherical.

Pygidium with slightly crenulated margins (hardly visible even under stereomicroscope but clearly visible under SEM).

##### Methyl green staining pattern.

Branchiae, lips and base of tentacles not stained. Extremity of tentacles staining and retained as blue/brown even after being washed in ethanol for some days (Fig. 2B, C). Thorax until segment XX, strongly staining ventrally, moderately laterally and poorly dorsally. Ventral stain on all shields, anterior half of each shield staining deeply, posterior part not staining (Fig. 2B, C). Anterior abdomen not coloured and posterior abdomen staining ventrally and dorsally with anterior half of each segment staining deeply, posterior part not staining; increasing colouration towards pygidium (Fig. 2B).

##### Morphological variation.

Complete individuals ranging from 9.0 to 22.7 mm in length, 0.5 to 1.1 mm in width at segment X and between 59 to 72 segments. Thoracic lengths vary between 3.1 and 7.8 mm. One gravid specimen was found (BAN.Pista.09). It was incomplete, but thoracic length was 5.1 mm and anterior width 0.7 mm. These measurements correspond to a small size species. Live specimens pinkish with translucent buccal tentacles; ventral shields divided in two parts, anterior part pinkish, posterior part blood red (Fig. 2A). Preserved specimens pinkish with ventral shields divided transversally in two parts. Crenulation of ventral collar of segment II is difficult to see under the binocular and not always visible under SEM. It probably depends on the contraction of the animal. All specimens, regardless of size, with a single pair of branchiae, one up to twice as long as the other (Fig. 2D). Eleven of the twenty observed specimens had the long branchiae on the right side. Number of anterior thoracic uncinigers with long-handled uncini is variable (from 2 to 9). This difference seems to not be dependent upon size. Nephridial papillae not always visible.

##### Etymology.

The name of species is dedicated to the nephew of the first author Colin Labrune who is already a little budding naturalist.

##### Type locality.

Only known from Banyuls-sur-Mer harbour, France (Mediterranean Sea).

##### Ecological notes.

*Pistacolini* sp. n. was sampled at 3 m depth on gravelly sand recently deposited manually in Banyuls-sur-Mer harbour. It was found in very high densities (446 ind. m^-2^ in April 2012 and 1176 ind. m^-2^ in July 2012) a few weeks after the sediments had been deposited. We sampled again in November 2012 but there was no more gravel and *Pistacolini* sp. n. was absent. The species is not found in the harbour if no gravel deposits are present. In the undisturbed part of the harbour, median granulometry was ca. 50 µm while the median granulometry of the gravelly sand in which this species is found was ca. 800 µm. In July 2017, we sampled a week after another fresh load of sediment with gravel had been deposited and we found high densities of *Pistacolini* sp. n. living in tubes made from heterogeneous sediment agglomerated with mucus.

##### Genetic data.

The COI gene was successfully sequenced and published at NCBI GenBank for paratype MNHN-IA-TYPE 1853 with accession number MK584933.

##### Remarks and discussion.

The presence of a single pair of branchiae is a stable character in *Pistacolini* sp. n. More than 100 specimens were observed, of different sizes and all of them had a single pair of branchiae, some were also observed alive. Our observations support Hutchings et al. (2017) but are not in agreement with Saphronova and Jirkov (2001), who hypothesised that this character is size-related. A detailed morphological and molecular study needs to be performed in order to investigate this hypothesis across a range of species with varying number of pairs of branchiae.

Although Gil (2011) mentioned *Pistacristata* as having only one pair of branchiae, based on Saphronova (1988), we considered that this species has two pairs of branchiae. The absence of consensus on this species does not have any consequence for this new species which always had some long-handled thoracic uncini, whereas Gil (2011) records *P.cristata* as lacking such long-handled even on large specimens. Among the seven species with a single pair of branchiae, there is no possible confusion of *P.colini* sp. n. with *Pistamirabilis* and with *P.spinifera* as both lack plumose branchiae. *Pistacolini* sp. n. is close to *P.adriatica* sharing the following characters: one pair of unequal sized plumose branchiae on segment II and presence of lateral lobes on segments II and III, lacking on segment IV. However, segment II of *P.adriatica* presents narrow lateral lobes while in *P.colini* sp. n. these lateral lobes are well developed. Lateral lobes of segment III are rectangular in *P.adriatica* rather than being asymmetrical and slightly displaced dorsally as in *P.colini* sp. n. Furthermore *P.colini* sp. n. can be differentiated by the absence of glandular ridges on segments II and III, which are present in *P.adriatica*. According to Mikac and Hutchings (2017), *P.godfroyi* and *P.dibranchis* which also have a single pair of branchiae, should be transferred to *Pistella*Hartmann-Schröder 1996 because they lack long-handled uncini. Therefore, they cannot be confused with *P.colini* sp. n. The lack of long-handled uncini is also the case for *Pistalornensis.* Furthermore, when first describing *Pistalornensis*, Pearson (1969) reported two obvious ligaments, one attached below the rostrum and the largest to the posterior basal corner of the uncini. These filaments are not present in *P.colini* sp. n.

According to Gil (2011), *Pistabansei* is the only *Pista* species in Europe to present one pair of “pompom like” branchiae and anterior long-handled uncini. The original description by Saphronova (1988) is based on an incomplete holotype with 16 segments, 3.2 mm wide collected at 105 m in Strait of Tartar, the Sea of Japan, north-western Pacific Ocean, and four damaged paratypes from the same locality. She also designated another eight paratypes (R/V “Vityaz” St 1576, 60°03'N, 168°46'E, 230 m, Olutorsky Bay, off Kamchatka Peninsula, Bering Sea, north-western Pacific Ocean) and 1 paratype (R/V “Sevastopol” St 1086, 495 m, 62°56'N, 9°19'W, between Iceland and Faroe Islands, North Atlantic Ocean), the material is deposited in Zoological Museum of Moscow State University. She also lists additional specimens not designated as type material from localities such as Davis Strait, Norwegian, Kara Sea (off Novaya Zemlya), White Sea in the North Atlantic, and Arctic Oceans, as well as Sea of Japan, Sea of Okhotsk, and Bering Sea in the north-western Pacific Ocean in depths of 120–606 m. Such a wide distribution is highly unlikely and we suggest that *P.bansei* sensu stricto is restricted to the north-western Pacific Ocean, while the rest of the material, including the one paratype from the North Atlantic Ocean represents another species, most likely part of the same species complex. Although, much of the material in Zoological Museum of Moscow State University is in poor condition, it most certainly belongs to multiple species. Therefore, Saphronova’s (1988) hypothesis that only adults have anterior thoracic uncini with well-developed handles, while such handles are absent in juveniles, cannot be accepted. Furthermore, her diagrammatic illustrations indicate neither the sizes of individuals nor where the specimens were collected.

All the specimens of *P.colini* sp. n. examined here, even the smallest (59 chaetigers, thoracic width at segment X: 0.5 mm), which are comparable in size with the individuals that Saphronova (1988) identified as juveniles (width between 0.4 and 1.15mm), had well-developed long-handled uncini, at least on chaetigers 1 and 2. Furthermore, in the original description, Saphronova (1988) described *P.bansei* with (1) an upper lip high and narrow while upper lip of *P.colini* sp. n. is large and rounded (2) large lateral lobes, positioned vertically and connected mid ventrally by a wide fold, although the connection between the two lateral lobes in *P.colini* sp. n. does not form a fold and does not look like the illustration in Saphronova (1988, fig. 8g–i). Moreover, Hilbig (2000) reports *P.bansei* with (1) moderate numbers of tentacles, usually broken off, although all specimens of *P.colini* sp. n. had some short and some long tentacles, rarely broken and (2) glandular ridges on segments II and III, which were not observed in *P.colini* sp. n. Furthermore, based on the holotype 1/47667, paratypes, and several additional specimens, Jirkov and Leontovich (2017) reported the presence of small lateral lobes on segment I in *P.bansei* which are not observed in *P.colini* sp. n. Therefore, *P.colini* sp. n., while similar to *P.bansei* in a number of characters, differs by the presence of long-handled uncini, even in the smallest specimens, and the fact that no glandular ridge was observed on segments II and III. Furthermore, the type locality of *P.bansei* is from the northern Pacific in cold deeper water (105 m).

Based on examination of the type material of Saphronova (1988) in Moscow and St Petersburg museums by Hutchings in 2018 (see Comparative material), *Pistabansei* is a North Pacific species currently only known with certainty from Tartar Strait and therefore, its range cannot overlap with that of any Mediterranean species. For these reasons, we describe *P.colini* as a new species from the Mediterranean Sea. Finally, this paper reinforces the need for a complete revision of the group of terebellids with long-handled uncini using both molecular and morphological data, especially those species with only a single pair of branchiae.

## Supplementary Material

XML Treatment for Pista

XML Treatment for Pista colini
